# Supplementary material for: Muvi schmallenbergi gen. nov., sp. nov. (Crustacea, Tanaidacea) from the southeast Australian coast, with comments on the distribution and habitat preferences of Chondropodinae
Source: PeerJ. 2021 Aug 11;9:e11607. doi: 10.7717/peerj.11607 (PMC8364323; doi:10.7717/peerj.11607)
Supplement: Supplemental Information 1 [file peerj-09-11607-s001.doc]

Specimens are deposited in Melbourne Museum (NMV, Australia).

Holotype female with accession number: MNV J74649.

Paratpe female with accesion number: MNV J74648.
